# A case of cerebral autosomal dominant arteriopathy with subcortical infarcts and leukoencephalopathy (CADASIL) presenting as post-infectious manifestation of SARS-CoV-2 infection

**DOI:** 10.1259/bjrcr.20210020

**Published:** 2021-04-09

**Authors:** Ishwariya Rajendran, Madhu Dutta Natarajan, Pooja Narwani, Omran Alzouabi, Khalil Kawafi, Navin Khanna

**Affiliations:** 1 Radiology fellow, Department of Radiology, Royal Oldham hospital, Pennine Acute NHS Foundation Trust, Oldham, United Kingdom; 2 Consultant Radiologist, Department of Radiology, Royal Oldham hospital Pennine Acute NHS Foundation Trust, Oldham, United Kingdom; 3 Stroke physician. Stroke Unit. Fairfield General Hospital. Pennine Acute Hospital NHS Foundation Trust, Oldham, United Kingdom; 4 Clinical Director of stroke unit. Stroke Unit. Fairfield General Hospital. Pennine Acute Hospitals NHS Foundation Trust, Oldham, United Kingdom; 5 Consultant Radiologist, Department of Radiology, Royal Oldham hospital. Pennine Acute NHS Foundation Trust, Oldham, United Kingdom

## Abstract

White matter hyperintensities (WMHs) lacunar infarcts and cerebral microbleeds are well-established features associated with cerebral autosomal dominant arteriopathy with subcortical infarcts and leukoencephalopathy (CADASIL). Increasing case reports and series recounts a wide array of neurological manifestations of COVID-19 including acute cerebrovascular disease, encephalopathy, encephalitis and demyelination. Recently association between COVID-19 and CADASIL has been identified. We describe an unusual case of CADASIL diagnosed as a possible post-infectious manifestation of COVID-19 patient with imaging features closely resembling post-infectious encephalomyelitis.

## Case presentation

A 45-year female presented with dysarthria and without any other neurological deficit. This led to suspicion of cerebrovascular accident, and the patient was admitted in the stroke ward. She previously experienced symptoms of COVID-19 such as fever, persistent cough, tiredness and generalised myalgia, and was self-isolating for past 14 days. However, at the time of presentation she was asymptomatic for COVID-19. Imaging and laboratory investigations reveal a potential diagnosis.

## Investigations

**Figure 1.**
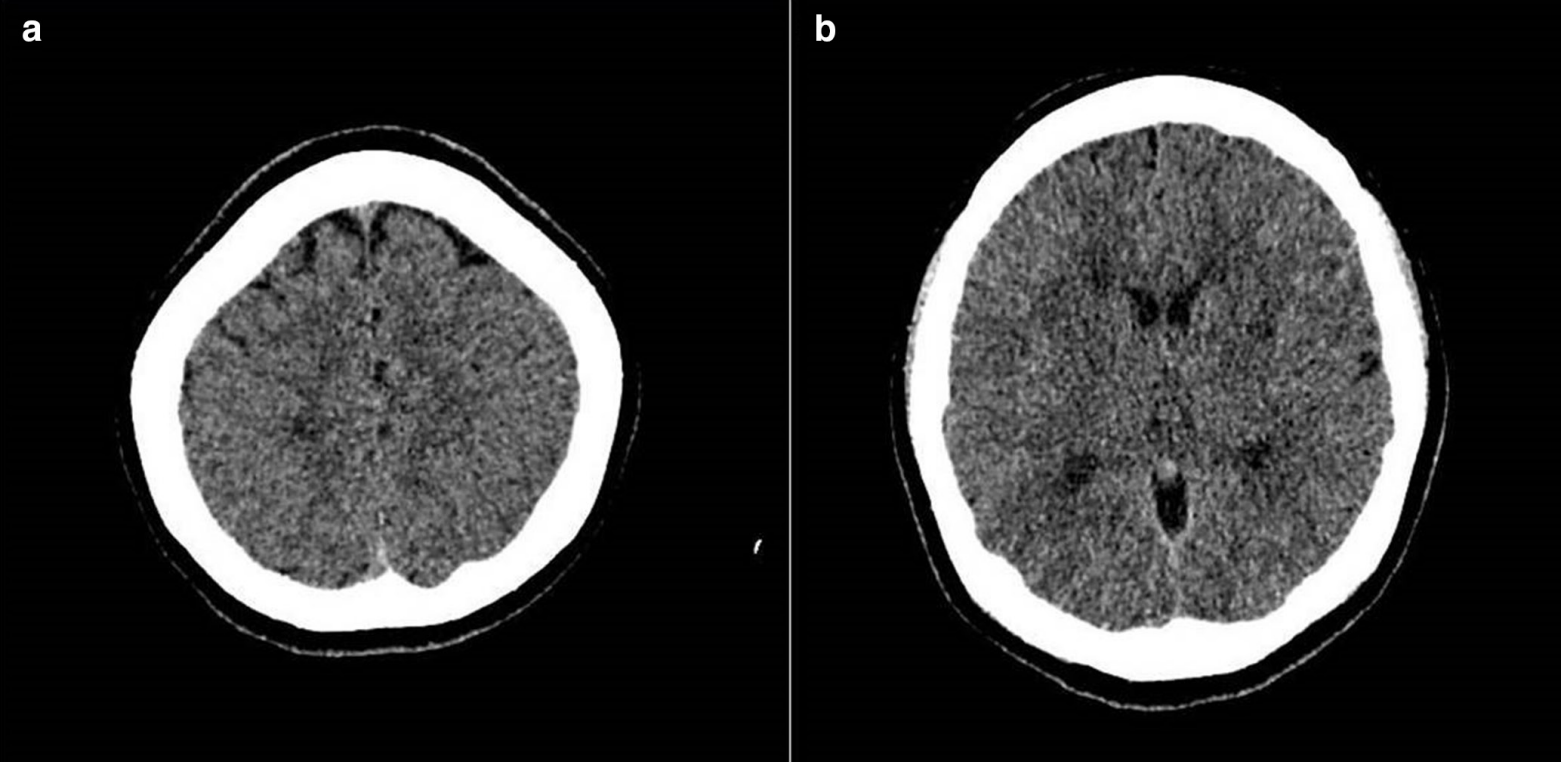
Axial non-contrast CT brain A and B demonstrate multiple hypodense lesions in bilateral cerebral hemispheres.

A non-contrast CT brain was performed, which revealed small white matter hypodensities in bilateral cerebral cortices (Figure 1). This was initially thought to be infarcts.

**Figure 2.**
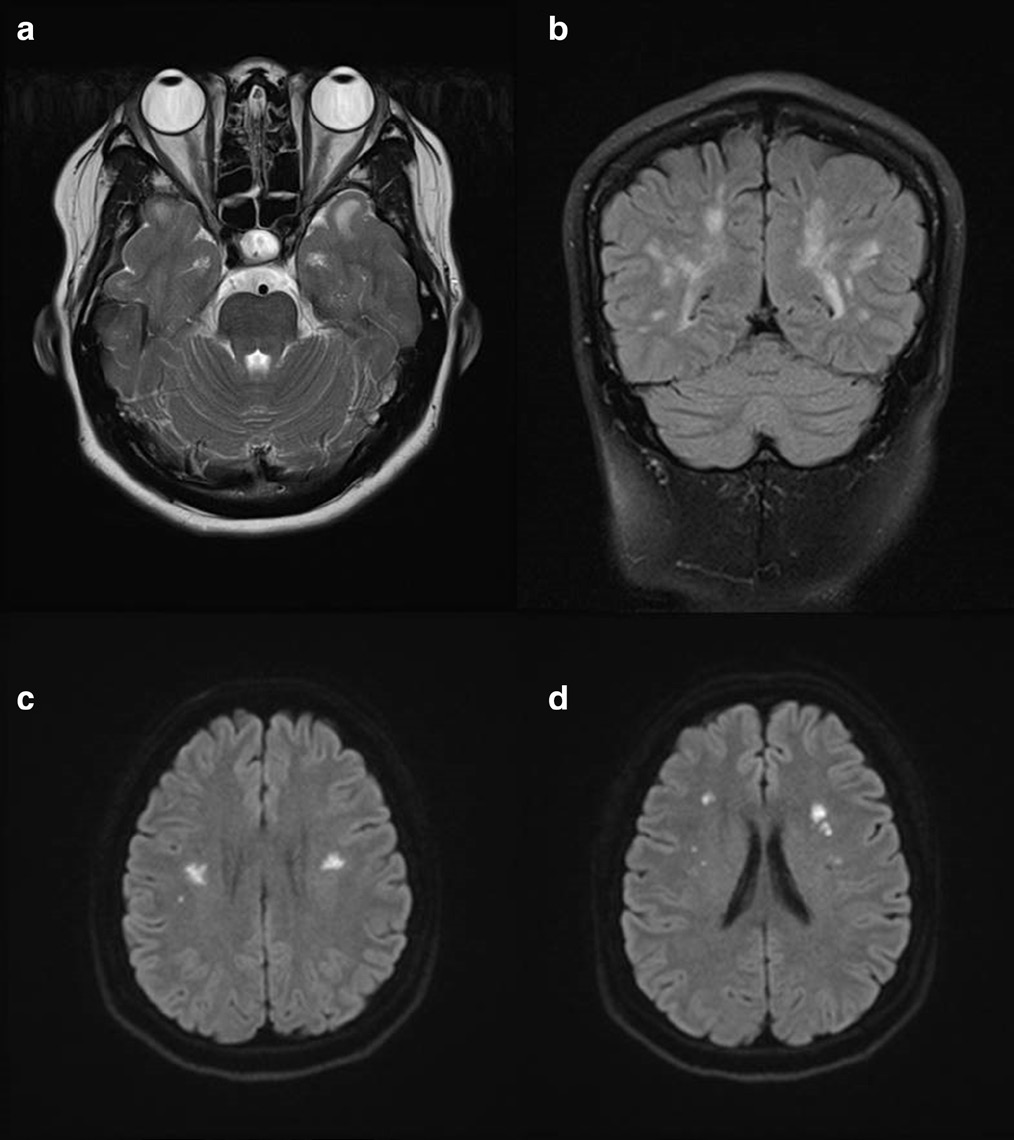
Non-contrast MRI brain axial *T_2_* weighted and coronal FLAIR MR images A) and B) Differentially involved anterior and posterior temporal white matter with symmetric distributions. Axial DWI MR images C) and D) Bilateral acute infarcts involving centrum semiovale and corona radiata.

MRI brain was further performed without contrast to evaluate the nature of these hypodensities. It revealed acute infarcts involving bilateral centrum semiovale and corona radiata. Widespread confluent bilateral subcortical and periventricular white matter hyperintensities were seen, with prominent involvement of anterior temporal lobe (Figure 2)). Chronic lacunar infarcts were also seen in right centrum semiovale and left basal ganglia.

Given the nature of multiple acute internal border zone infarcts and chronic infarcts with a background of anterior temporal lobe predominant white matter disease, a possibility of CADASIL and vasculitis was raised. Also, other causes of stroke involving multiple arterial territories in young patients were sought. Although not currently symptomatic for COVID-19, in view of the previous symptoms, a nasopharyngeal swab was done, which revealed a positive SARS-CoV-2 by PCR. The patient had CT-angiogram brain to look for vasculitis, which did not reveal any abnormality. The CRP was normal, 3.1 mg l^−1^. Screening for other causes of stroke in young people was done, including antinuclear antibody, ANCA, anti-DS DNA, anti-CCP, antibody to cardiolipin, prothrombin gene variant, factor V Leiden mutation, which were negative. Infectious disease screening for CMV, Hepatitis B and C, HSV, Treponema and HIV also turned negative. Samples were sent for NOTCH three gene testing.

**Figure 3.**
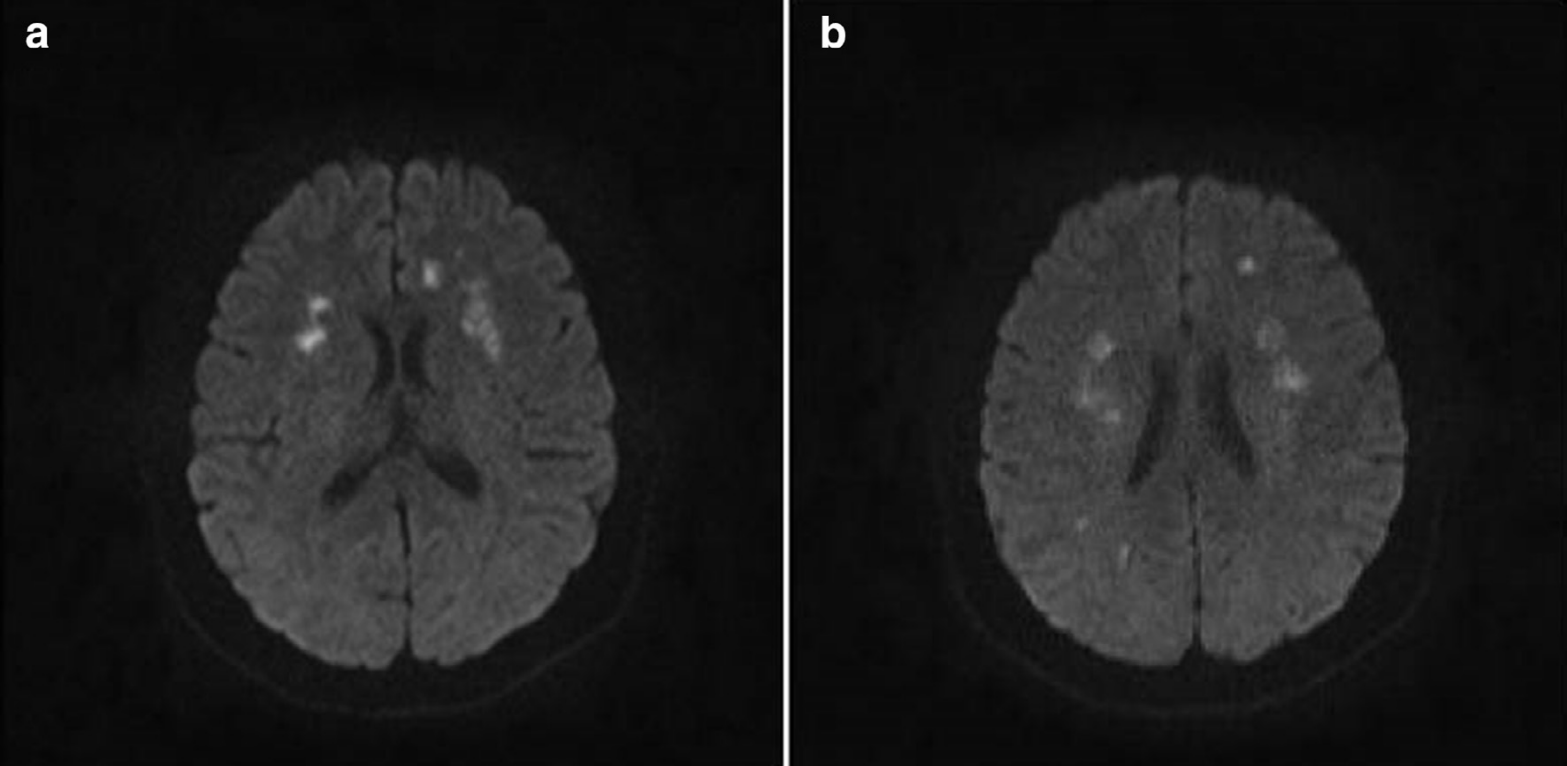
Axial DWI MR images A and B shows acute infarcts in bilateral corona radiata.

Eight days after the initial episode, she developed new acute confusion. MRI brain and whole spine with contrast was performed, which demonstrated interval new infarcts in bilateral corona radiata (Figure 3). No abnormal enhancement was observed in the brain, and no abnormality was seen in the spine. Lumbar puncture was done, which was negative for HSV, VZV, adenovirus, enterovirus and parechovirus with normal CSF total protein, glucose and no oligoclonal bands. CSF PCR screen for SARS-CoV-2 was done, which turned negative. Interestingly, IgG4 subclass was increased (1.220 g l^−1^). Other extractable nuclear antigens were negative.

## Differential diagnosis

In the light of evolving neurological manifestations of COVID-19, acute stroke can be a direct manifestation of COVID-19. The principal differential diagnosis includes demyelinating and vascular diseases. The other differentials considered in this patient were multiple sclerosis, acute demyelinating encephalomyelitis (ADEM) or vasculitis related to Covid-19 infection.

## Treatment

Considering the rapid interval progression of infarcts, a provisional diagnosis of central nervous sytem vasculitis or CADASIL triggered by COVID-19 was made. Methylprednisolone was started 500 mg for 3 days, followed by prednisolone 40 mg OD for four weeks. Patient symptoms improved and was discharged.

## Outcome and follow up

A follow-up MRI done seven weeks later showed the disappearance of initial DWI and ADC changes. The genetic investigations proved that the patient is heterozygous for a pathogenic NOTCH3 missense variant. There was no known family history of CADASIL at presentation. Her father had an episode of a stroke at the age of 52, resulting in permanent right-sided weakness.

## Discussion

CADASIL is the most frequent single-gene disorder of small cerebral arteries, resulting from *NOTCH3 mutations*, which is more commonly expressed in vascular smooth muscle cells, causing deposition of granular and osmiophilic substances within the vascular smooth muscle cell membrane.^1^ This gradually leads on to fibrosis and small infarcts in the white matter, deep grey matter and the pons. The diagnosis of CADASIL is based on imaging features and laboratory examinations. The classical diagnostic features includes leukoaraiosis predominantly involving anterior parts of the temporal white matter, the periventricular, posterior temporal white matter and multiple, bilateral small infarcts involving deep white matter, basal ganglia, thalamus and pons. Electron microscopic examination of vascular smooth muscles in the brain, skeletal muscle, peripheral nerves and skin can demonstrate granular and osmiophilic substance layers. Diagnosis of CADASIL is confirmed by detecting NOTCH three mutations by DNA analysis.^2^

As of 6 December, COVID-19 has resulted in over 65.8 million cases reported and 1.5 million deaths globally since the beginning of the pandemic.^3^ Although the usual presentation of COVID-19 is an upper respiratory infection or flu-like symptoms, it can potentially affect multiple organs. The neurological manifestation of COVID-19 is being widely recognised and constantly evolving, which could be direct effects of the infection, para-infectious or post-infectious inflammation of nervous system and vasculature or secondary to systemic disease.^4^

The hypercoagulable state in COVID-19 and many contributing factors related to acute inflammatory response as indicated by increased interleukin and C-reactive protein has led to cerebrovascular disease (CVD) as the most common neurological presentation in COVID-19. Strong early recognition and timely management of CVD in COVID-19 patients can reduce morbidity and mortality. Acute manifestations also includes venous sinus thrombosis, large vessel occlusion and intracranial haemorrhage. The neurological manifestations attributed to direct neuroinvasion are relatively rare compared to secondary sequelae to increased inflammatory response, which can impair endothelial function and trigger para-infectious autoimmune response.

Post-infectious autoimmunity has been recognised in COVID-19, causing neurological manifestations including acute necrotising encephalopathy (ANE) and acute disseminated encephalomyelitis (ADEM). ANE is rarely attributed to COVID-19, which can cause multifocal symmetric involvement of grey and white matter with characteristic involvement of bilateral thalami. ADEM, which tends to be a monophasic demyelinating disorder due to perivenular inflammation can present with multiple reversible white matter hyperintense lesions in brain and spine, with frequent involvement of subcortical grey matter. The lesions in ADEM tend to be larger and rounded with prominent involvement of deep grey matter nuclei, thalamus, and brain stem with peripheral rim enhancement as in other demyelination conditions or disorders or lesions. Inflammatory vasculopathy associated with COVID-19 infection can show irregular narrowing of arteries on CT angiogram.^5^

Another rare neurological disorder in COVID-19 infection is inflammatory leukoencephalopathy, which is attributed to a para-infectious or autoimmune response. The imaging features are non-specific and include demyelinating lesions which are often ovoid or ring like involving periventricular white matter and bulbomedullary junction along with scattered microhemorrhage. It has been emphasised that COVID-19 may act as an infective trigger similar to Ebstein Barr Virus in multiple sclerosis.^6^

Multiple sclerosis has a typical involvement of corpus callosum, U- fibres, arising from callososeptal interface, brain stem, cerebellum and spinal cord. Active lesions show post-contrast enhancement. In vascular diseases, focal white matter hyperintensities are identified in the deep gray matter structures, corona radiata and semioval center. Anterior temporal pole involvement is rare in sporadic small vessel disease. The presence of callosal commissure lesions helps to differentiate CADASIL from small-vessel disease.^2^

This rare case demonstrates a unique presentation of CADASIL as a possible post-infectious neurological manifestation of COVID-19 in a patient with no known familial inheritance. Anterior temporal lobe predominant white matter hyperintensities, lacunar infarcts involving centrum semiovale, thalamus, basal ganglia and pons and cortical microbleed outside the ischemic region are classic imaging features, which can narrow down the accurate diagnosis. More importantly, the presence of chronic lacunar infarcts and specific pattern of white matter changes provided an important clue for the underlying condition.

At the time of diagnosis (April 2020), there were no published case reports. However, at present, there are three case reports citing the association between CADASIL and COVID. But, there are a few unique features in this case which are:

- This is post-infectious manifestation of SARS CoV-2
- Completely worked out with all relevant investigations
- No known familial inheritance

The importance of this case is in establishing the wider neurological manifestations of SARS CoV-2 and in understanding the pathogenesis. With COVID-19 becoming rampant, such neurological manifestations could be mere coincidence rather than SARS-CoV-2 being a precipitating agent, which is difficult to conclude from a single-case reports. However, to establish clear association between CADASIL and COVID-19, many such case reports or case series are needed.

## Learning points

1. The case illustrates the importance of identifying the pattern of involvement to arrive at the diagnosis of CADASIL, in contrast to other neurological manifestations of COVID-19.
2. The association of CADASIL exacerbation by COVID-19 reinforces the pathophysiology related to endothelial injury behind the CVD precipitated by COVID-19, which also can be a potential infectious trigger here in precipitating the disease.
3. This case throws light on the post-infectious neurological presentation in COVID-19, which could be due to the inflammatory response. This raises the concern for identifying such patients and advocates early use of anti inflammatory agents for improved outcome.
4. Timely diagnosis and appropriate management of the condition can improve the long-term outcome of the patients.
5. We recommend COVID-19 screening in patients presenting with new-onset CVD to detect post-infectious manifestations of COVID-19.
